# Type 5 diabetes mellitus: nutritional-imprinted β-cell insufficiency, diagnostic gaps, and emerging therapeutic strategies

**DOI:** 10.3389/fendo.2026.1739521

**Published:** 2026-03-16

**Authors:** Shida Chen, Ming Lu

**Affiliations:** Department of Endocrinology, The Second People’s Hospital of Bengbu City, Bengbu, China

**Keywords:** endocrine–nutritional interaction, lean diabetes phenotype, malnutrition-related diabetes, nutritional metabolism, nutritional rehabilitation therapy, precision classification, type 5 diabetes mellitus, β-cell reserve

## Abstract

Historical descriptions of malnutrition-related diabetes mellitus (MRDM) have regained attention in contemporary discourse, with the term type 5 diabetes mellitus (T5DM) increasingly used as a harmonized research construct to describe insulin-deficient diabetes associated with early-life undernutrition. Although the *International Classification of Diseases, 11th Revision* (ICD-11) includes categories related to malnutrition-associated diabetes, the pathophysiological interpretation, diagnostic boundaries, and therapeutic implications of T5DM remain incompletely defined, resulting in variable clinical adoption. Consequently, many affected individuals continue to be classified as lean type 2 diabetes and managed within obesity-centric care paradigms. This narrative review synthesizes existing evidence to advance a conceptual framework that distinguishes T5DM from other lean diabetes phenotypes by integrating developmental nutritional reserve, β-cell functional capacity, and autoimmune status. Clinical observations are highlighted to indicate that body size–based descriptors alone may not adequately capture clinically relevant nutritional heterogeneity. Emerging pharmacological evidence is also reviewed, suggesting that responses to glucagon-like peptide-1 receptor agonists, sodium–glucose cotransporter-2 inhibitors, and insulin secretagogues may not be determined solely by residual β-cell functional reserve, but may also be modulated by underlying nutritional status. Building on this premise, a nutrition-integrated pharmacometabolic model is proposed as a hypothesis-generating framework, in which standardized nutritional assessment is considered alongside β-cell evaluation to inform exploratory treatment stratification rather than prescriptive clinical guidance. Finally, priorities for translational research are outlined, including the development of consensus-based indicators of nutritional reserve, improved phenotypic classification frameworks, and nutritional reserve–stratified randomized trials. Conceptualizing T5DM as a nutritionally conditioned metabolic phenotype may facilitate more targeted research agendas and support context-sensitive, individualized interventions in populations where malnutrition and diabetes intersect.

## 1 Introduction

Increasing recognition that diabetes comprises a spectrum of heterogeneous metabolic phenotypes, rather than a single glycemic disorder, has highlighted important limitations of traditional binary classification frameworks (1). Although advances in precision endocrinology have motivated more nuanced taxonomies, contemporary classification systems remain largely structured around obesity-associated insulin resistance (IR) and autoimmune-mediated β-cell destruction (2). In contrast, diabetes presentations associated with early-life undernutrition and adverse developmental environments remain under-represented within prevailing clinical and research paradigms (3).

The long-standing dichotomy separating type 1 diabetes mellitus (T1DM) from type 2 diabetes mellitus (T2DM) does not fully capture the metabolic diversity observed across global populations (1, 2). This limitation is particularly evident in undernourished settings, where relative β-cell insufficiency has been reported in the absence of obesity or overt autoimmunity (3, 4). Recognition of such heterogeneity has historically prompted efforts to refine classification beyond the conventional T1DM–T2DM framework, including latent autoimmune diabetes in adults (LADA), maturity-onset diabetes of the young (MODY), and diabetes secondary to systemic disease or pharmacological exposure (2).

Historical descriptions of malnutrition-related diabetes mellitus (MRDM) characterized predominantly young individuals with low BMI who presented with insulin-requiring hyperglycemia, relatively low reported propensity for ketosis, and limited evidence of autoimmunity—features that did not align neatly with classical T1DM or T2DM definitions (3, 5). Rather than introducing a novel nosological entity, the contemporary term “type 5 diabetes mellitus” (T5DM) has been proposed as a harmonized research construct intended to integrate these historical observations with emerging data on insulin-deficient diabetes in nutritionally vulnerable populations (3, 5, 6). In this manuscript, historical descriptions are referred to as malnutrition-related diabetes mellitus (MRDM), recent consensus terminology as undernutrition-associated diabetes, and the term “T5DM” is used as a harmonized research construct unless otherwise specified.

**Figure 1:**
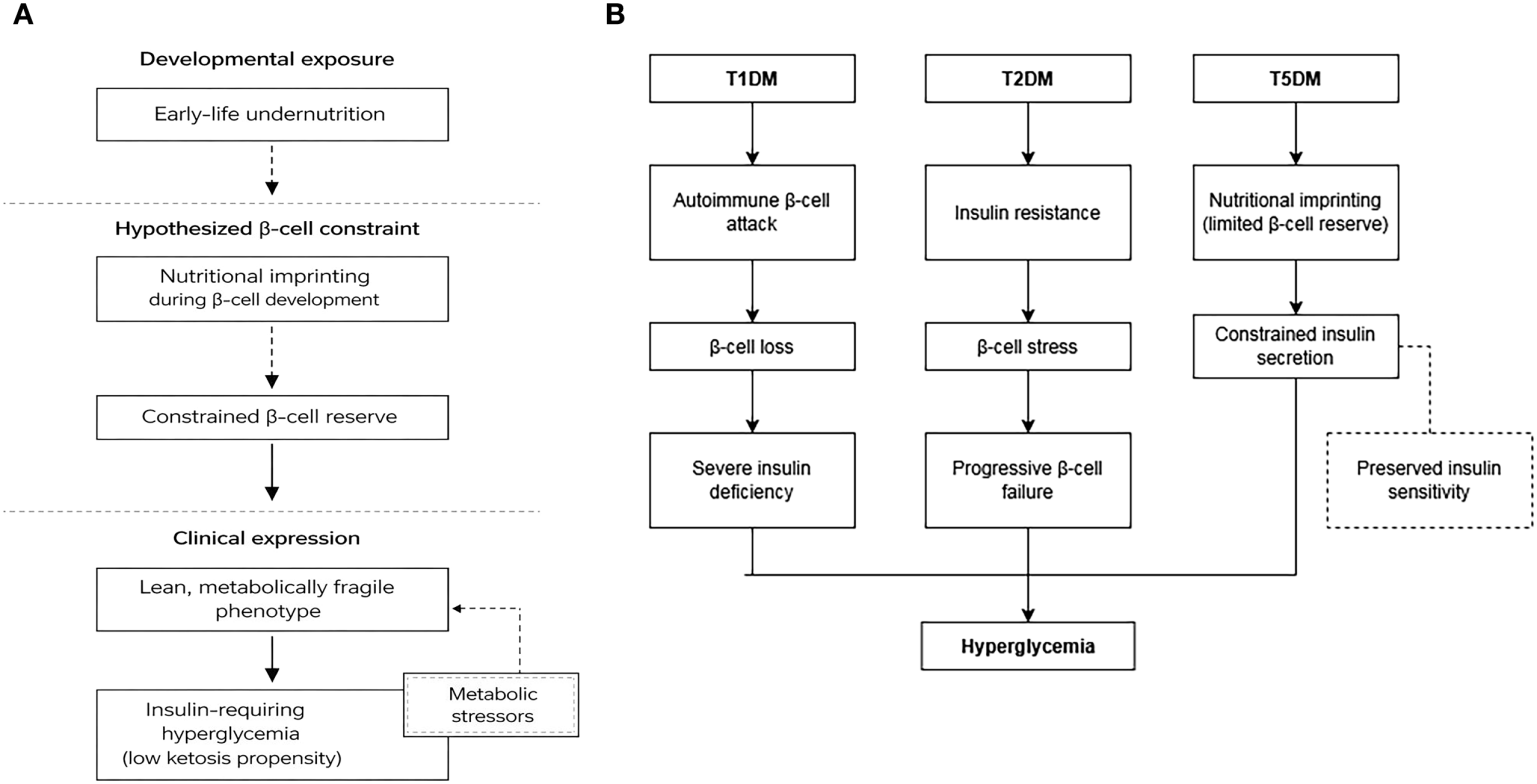
**(A)** Conceptual framework linking early-life undernutrition to β-cell–limited diabetes phenotypes. This schematic depicts a hypothesized sequence whereby early-life undernutrition may imprint pancreatic β-cell development, resulting in a constrained β-cell reserve and a lean, metabolically fragile phenotype. Under subsequent metabolic stressors (e.g., intercurrent illness, infection, or nutritional transition), limited β-cell reserve may manifest as insulin-requiring hyperglycemia, often with a lower propensity for ketosis in reported cohorts. This conceptual framework highlights nutrition-conditioned β-cell vulnerability and distinguishes it from autoimmune β-cell destruction in T1DM and insulin resistance–dominant mechanisms in obesity-associated T2DM. **(B)** Comparative schematic of metabolic pathways leading to hyperglycemia in T1DM, T2DM, and T5DM. This schematic contrasts three conceptual trajectories that converge on hyperglycemia but differ in upstream drivers. In T1DM, autoimmune β-cell attack leads to β-cell loss and severe insulin deficiency. In obesity-associated T2DM, insulin resistance predominates, accompanied by β-cell stress and progressive β-cell failure. In the proposed T5DM framework, early-life undernutrition is hypothesized to imprint β-cell developmental capacity, resulting in limited β-cell reserve and constrained insulin secretion, with relatively preserved insulin sensitivity.

Within this conceptual framework, early-life nutritional deprivation is hypothesized to constrain β-cell developmental capacity and reduce functional reserve; however, this interpretation remains provisional and requires further empirical validation. The notion of “nutritional imprinting,” illustrated schematically in **Figure 1A**, is therefore presented as a heuristic model rather than a definitive mechanistic explanation. A comparative schematic contrasting autoimmune β-cell destruction (T1DM), insulin resistance–dominant metabolic overload (T2DM), and nutrition-conditioned limitation of β-cell reserve (proposed T5DM) is shown in **Figure 1B**, highlighting differences in upstream drivers despite convergence on hyperglycemia.

Observational studies and experimental models suggest that sustained protein–energy deficiency during critical developmental windows may be associated with reduced β-cell mass and diminished insulin secretory capacity, creating a metabolically vulnerable endocrine state (6, 7). Some reports indicate that such impairments may persist despite later nutritional improvement, although the magnitude and clinical relevance of this persistence remain incompletely defined (6, 7). In contrast to obesity-associated T2DM—where IR driven by adiposity-related inflammation predominates—presentations consistent with the proposed T5DM framework are characterized by relative β-cell insufficiency with comparatively preserved insulin sensitivity (2, 8). Direct human evidence demonstrating a permanent structural β-cell deficit attributable solely to early-life undernutrition, however, remains limited.

Conventional anthropometric indices have limited sensitivity for detecting clinically meaningful nutritional vulnerability in low-BMI diabetes presentations (9–11). Accumulating evidence further indicates that impaired nutritional status is associated with increased complication burden and mortality among individuals with diabetes, underscoring the prognostic relevance of nutritional factors not captured by body size alone (12–15).

Despite growing recognition of phenotypic heterogeneity, current proposals related to T5DM remain largely conceptual, and standardized operational diagnostic criteria have not been universally established (2, 3, 5). In routine practice, classification frequently relies on readily available anthropometric descriptors, which may inadequately reflect endocrine–nutritional vulnerability in insulin-deficient, nutritionally conditioned presentations (2, 6). Consequently, such phenotypes may remain under-recognized, limiting opportunities for earlier identification of individuals at risk of accelerated β-cell functional deterioration (4–6). This review therefore synthesizes current evidence related to T5DM, integrating epidemiological observations, diagnostic challenges, and nutritional pathophysiological insights to provide a structured research-oriented framework rather than formal diagnostic or therapeutic guidance (3–5).

This manuscript is a narrative review. Literature was identified through structured searches of major biomedical databases, including PubMed and Embase, over the past decade using combinations of terms related to MRDM, undernutrition-associated diabetes, β-cell function, and diabetes classification. Reference lists of key reviews and consensus statements were manually screened for additional relevant studies. Given the heterogeneity of available evidence, formal quantitative synthesis and uniform risk-of-bias assessment were not undertaken, and findings are interpreted in a conceptual, hypothesis-generating context.

## 2 Definition and characteristics of T5DM

### 2.1 Historical evolution: J-type diabetes → MRDM → ICD-11 malnutrition-associated diabetes → current type 5 construct

**Figure 2:**
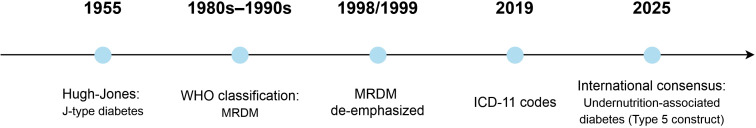
Historical classification trajectory of undernutrition-associated diabetes. The timeline highlights major milestones from the initial description of “J-type” diabetes (1955), through WHO classification discussions of MRDM (1980s–1990s) and its subsequent de-emphasis (1998/1999), to ICD-11 coding (2019) and the 2025 international consensus defining the contemporary Type 5 construct.

The phenotype currently discussed as type 5 diabetes mellitus (T5DM) is best understood as a contemporary research construct that consolidates multiple historical descriptions of insulin-deficient diabetes occurring in lean individuals with exposure to undernutrition (1, 5, 6). Early reports from the mid-20th century, including Hugh-Jones’ report of “J-type” diabetes in Jamaica (1955), which described young, lean patients presenting with marked hyperglycemia, insulin requirement, and a reportedly lower propensity for ketosis than classic autoimmune type 1 diabetes (16). Subsequent reports across low- and middle-income settings described similar phenotypes variably labelled as tropical diabetes or malnutrition-related diabetes mellitus (MRDM) (17, 18). The key historical milestones are summarized in **Figure 2**.

In WHO classification discussions from the mid-1980s to the late 1990s, MRDM was incorporated as a proposed category; however, it remained controversial because operational boundaries were inconsistent and overlap with other entities (autoimmune diabetes, pancreatogenic diabetes, and lean presentations of type 2 diabetes) was substantial (19). This reappraisal coincided with increasing availability of diagnostic tools for autoimmunity, pancreatic disease, and monogenic diabetes, and MRDM was progressively deprioritized in later classification frameworks, reflecting concerns about reproducibility rather than a definitive refutation of the underlying phenotype (20).

In parallel, the International Classification of Diseases, 11th Revision (ICD-11) introduced coding categories relevant to malnutrition-associated diabetes, providing a pragmatic administrative scaffold but not resolving the biological or clinical boundaries of the condition (21). More recently (late 2010s–2025), renewed attention has been driven by contemporary cohorts describing atypical, lean, insulin-deficient diabetes and by international consensus efforts proposing undernutrition-associated diabetes as a distinct research focus (1, 4). In this context, the term “T5DM” functions as a unifying research label of phenotyping (nutritional reserve, β-cell reserve, and autoimmunity) and to reduce misclassification as lean type 2 diabetes (1, 5).

### 2.2 Contemporary definition and diagnostic boundaries

Although the historical label of malnutrition-related diabetes mellitus (MRDM) has been repeatedly invoked across decades, its diagnostic reproducibility has remained limited. A major challenge is the substantial phenotypic overlap with other forms of lean diabetes, including latent autoimmune diabetes in adults (LADA), pancreatogenic diabetes (type 3c), and insulin-deficient presentations of type 2 diabetes, particularly in settings where autoantibody testing, pancreatic imaging, and genetic evaluation are not routinely available (3–5).

Importantly, reliance on low BMI as a defining feature lacks specificity, as constitutional leanness, sarcopenia, and catabolic weight loss during uncontrolled diabetes may produce similar anthropometric profiles. Accordingly, anthropometry-based criteria alone are insufficient to distinguish nutritional imprinting from non-nutritional etiologies of insulin deficiency (3, 11).

Additional challenges arose from substantial phenotypic overlap with other lean diabetes entities, including LADA, pancreatogenic diabetes, and lean presentations of T2DM, particularly in the absence of validated markers capable of reliably differentiating nutritional reserve and pancreatic β-cell functional capacity (3–5).

Contemporary proposals framing T5DM as a provisional research construct therefore emphasize a multidimensional definition incorporating evidence of undernutrition exposure or reduced nutritional reserve, impaired β-cell secretory capacity, and exclusion of autoimmune diabetes. However, validated biomarkers and consensus operational criteria remain incomplete, reinforcing that T5DM should currently be regarded as a hypothesis-generating phenotype rather than an established clinical diagnosis (1, 5, 6).

### 2.3 Clinical manifestations

Within current conceptual frameworks, T5DM is described as a low-BMI, insulin-deficient presentation frequently associated with impaired endogenous insulin secretory capacity in the absence of islet autoimmunity (3, 4). Compared with classical T1DM, ketosis may be less frequent in some reported cohorts; however, this feature appears variable and should not be considered a defining characteristic (5, 6).

Nutritional depletion has been reported in individuals meeting proposed T5DM characteristics and may manifest as reduced muscle mass, unintentional weight loss, and micronutrient inadequacy, contributing to metabolic instability (9–11). Pancreatic exocrine insufficiency and gastrointestinal manifestations—such as early satiety, bloating, nausea, and delayed gastric emptying—have also been documented in subsets of patients with diabetes and may further impair nutrient intake and absorption, potentially exacerbating glycemic variability and protein–energy deficiency (22–26). In this context, structured nutritional assessment tools, including the Patient-Generated Subjective Global Assessment (PG-SGA), have been used to characterize nutritional status, with evidence suggesting that nutritional deficits may worsen alongside increasing metabolic stress and reduced physiological reserve (27).

Collectively, T5DM may be conceptualized as an integrated clinical phenotype in which lean body habitus alone is insufficient for accurate classification (1, 2). Recognition therefore often benefits from consideration of nutritional status, β-cell functional reserve, and careful differentiation from autoimmune or monogenic forms of diabetes, as reliance on glycemic indices or body size–based descriptors alone may increase the risk of misclassification as lean T2DM or other atypical presentations (1–4). These features, summarized across historical and contemporary cohorts, are provided in **Table 1**, highlighting the consistent clinical pattern of T5DM over time. Notably, systematic longitudinal outcome data were not reported in these cohorts, highlighting a persistent evidence gap across decades.

**Table 1:** Clinical phenotype associated with T5DM across historical and contemporary cohorts (1955–2024).

Study [Ref]/country/year	Age at diagnosis	BMI	Insulin dependence at presentation	Ketosis	C-peptide	Autoantibodies	Exocrine	Outcome
Hugh-Jones (16); Jamaica; 1955	Young	Lean	Required	Low/rare	NA	NA	NR	No longitudinal data
Abu-Bakare (17); Tropics; 1986	Young	Lean	Variable	Variable	NR	NR	NR	No longitudinal data
Chattopadhyay (18); India; 1995	Young	Lean	Required	Low/rare	Reduced	Negative	Mild EPI	No longitudinal outcomes
Lontchi-Yimagou (4); Multi; 2022	Young–mid	Lean	Required	Low/rare	Reduced	Negative	NR	Cross-sectional
Siddiqui (90); India; 2022	Young	Lean/normal	Variable	NR	Reduced	Negative	NR	Cross-sectional
Kibirige (91); SSA; 2024	Adult	Underweight	NR	NR	Reduced	Negative	NR	Cross-sectional

NA, not available due to historical or technical limitations; NR, not reported in the original publication; EPI, exocrine pancreatic insufficiency. C-peptide values are shown where reported; early MRDM cohorts predated standardized assays and are therefore indicated as NA rather than NR. Ketosis reflects qualitative descriptions in the original reports, as quantitative rates were infrequently provided. Longitudinal outcome data were not systematically reported in historical MRDM cohorts, and most contemporary phenotype-consistent studies were cross-sectional in design. Historical MRDM cohorts and contemporary phenotype-consistent insulin-deficient, non-autoimmune, low-BMI diabetes populations are included as representative of the clinical phenotype associated with the proposed construct of T5DM.

## 3 Diagnostic challenges

### 3.1 Ambiguity of diagnostic criteria

Although T5DM has increasingly been discussed as a nutritionally conditioned research phenotype, its clinical identification remains constrained by the absence of validated and operational diagnostic criteria that can be consistently applied across settings (3, 5, 6). This ambiguity partly reflects the historical origins of the concept: early descriptions of MRDM were largely derived from region-specific clinical observations rather than prospectively defined diagnostic frameworks, contributing to heterogeneous interpretations of insulin requirement, ketosis propensity, and disease boundaries (3, 5, 6).

While these descriptive features facilitated early recognition, subsequent clinical and epidemiological experience has demonstrated limited diagnostic specificity in practice (3, 5). Substantial phenotypic overlap with other low-BMI or insulin-deficient diabetes presentations—including atypical T2DM—continues to challenge phenotype-based classification and supports development of more operational approaches integrating nutritional status and β-cell functional assessment (3, 4).

Continued reliance on overly simplified anthropometric proxies has limited clinical applicability in nutritionally heterogeneous populations, particularly in settings where constitutional leanness is common and historical MRDM criteria are less transferable to contemporary presentations.

The absence of standardized operational criteria and validated biomarkers also limits consistent characterization of T5DM (3). Pancreatic exocrine dysfunction, a plausible accompaniment of chronic undernutrition, has been documented using heterogeneous methods in studies of diabetes and nutritional deficiency, but has not yet been systematically integrated into contemporary diabetes phenotyping frameworks (7, 25, 26). Non-uniform thresholds also contribute to variability in prevalence estimates and risk stratification across studies (9, 28–30).

These limitations indicate that descriptive or anthropometry-based criteria are insufficient, motivating reproducible, multidimensional diagnostic approaches that more accurately reflect contemporary understanding of diabetes pathophysiology and nutritional–metabolic heterogeneity (1, 3, 5, 11, 15, 31).

Given the persistent heterogeneity and historical diagnostic ambiguity surrounding T5DM, the absence of reproducible operational criteria has limited cross-study comparability and cohort harmonization. To facilitate standardized case identification in observational and interventional research, provisional operational research criteria for suspected T5DM are outlined in Box 1. These criteria are intended to support structured phenotyping and hypothesis-driven investigation and should not be interpreted as formal diagnostic standards.

#### 3.1.1 Box 1. provisional operational research framework for suspected T5DM

Scope: This framework is intended for research cohort identification and exploratory phenotyping. It does not constitute formal diagnostic criteria and should not replace established diabetes classification systems.

##### 3.1.1.1 Core criteria (all required)

- Confirmed diabetes mellitus according to ADA or WHO glycemic thresholds.
- Low or low-normal BMI (ethnicity-adjusted): Asian populations <23 kg/m²; non-Asian populations <25 kg/m². A stricter threshold <21 kg/m² may be considered in high-undernutrition settings.
- Evidence of nutritional vulnerability (≥1): documented childhood/adolescent undernutrition; nutritional risk by validated tools (e.g., CONUT ≥ mild risk; PG-SGA category B/C); or biochemical indicators of reduced nutritional reserve interpreted outside acute inflammatory states.

##### 3.1.1.2 Supportive metabolic features (≥2 recommended)

- Low–moderate fasting C-peptide (C-P) and/or reduced stimulated C-P response.
- Negative islet autoantibodies (GAD65, IA-2, ZnT8).
- No overt clinical features suggestive of insulin resistance, including marked central adiposity, acanthosis nigricans, or clinically apparent non-alcoholic fatty liver disease (NAFLD).
- Low-BMI presentation without prior obesity history.

##### 3.1.1.3 Exclusion criteria (any excludes classification)

- Chronic pancreatitis or fibrocalculous pancreatic diabetes.
- Established pancreatogenic (type 3c) diabetes.
- Suspected monogenic diabetes (e.g., early onset with autosomal dominant inheritance pattern).
- Chronic systemic glucocorticoid exposure.
- Classical autoimmune T1DM.

#### 3.1.2 Operational research classification

Individuals meeting all core criteria, ≥2 supportive metabolic features, and no exclusion criteria may be operationally classified as ‘suspected T5DM (research phenotype)’ for cohort enrichment and mechanistic investigation.

##### 3.1.2.1 Implementation considerations

- Interpret nutritional indices in clinical context, particularly in inflammatory, renal, or hepatic conditions.
- Stimulated C-P testing is preferred where feasible.
- Threshold calibration may require adaptation across populations undergoing nutritional transition.

### 3.2 Preliminary evaluation framework for suspected T5DM

**Figure 3:**
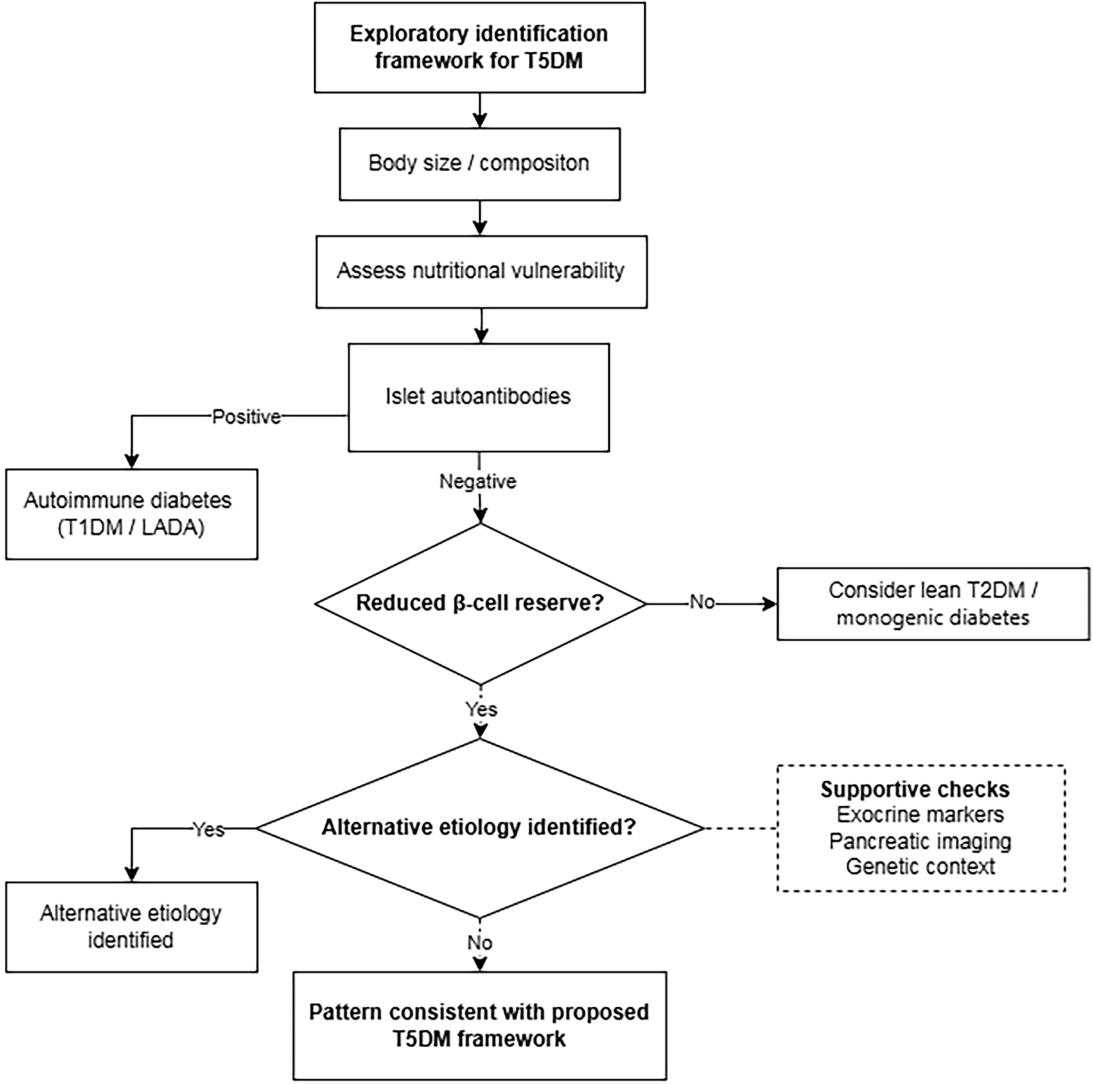
Exploratory identification framework for T5DM. Patients presenting with diabetes in a lean or atypical phenotype undergo stepwise evaluation to distinguish nutritional vulnerability from constitutional leanness. Following identification of nutritional impairment, negative islet autoantibody testing helps exclude autoimmune β-cell destruction characteristic of T1DM or LADA. Subsequent assessment of β-cell reserve and IR facilitates differentiation from IR-dominant lean T2DM. Supportive indicators—including pancreatic exocrine dysfunction, pancreatic imaging features, and family history with targeted screening for MODY—may assist in refining phenotypic classification. Individuals demonstrating nutritional impairment, evidence of reduced β-cell reserve, and absence of marked IR are considered consistent with the proposed T5DM research framework, underscoring the importance of nutrition-integrated diagnostic approaches.

Although a formal diagnostic algorithm for T5DM has not been established, a pragmatic, conceptually oriented evaluation framework may be cautiously inferred from existing evidence and expert discussions to support exploratory case identification in research settings (1, 3). This framework is not intended for routine diagnosis or clinical management and does not replace formal diagnostic criteria; rather, it aims to reduce misclassification (e.g., as low-BMI T2DM) and to facilitate earlier phenotype-oriented stratification. **Figure 3** outlines this exploratory identification framework, illustrating a stepwise approach integrating nutritional assessment, autoantibody evaluation, and β-cell functional testing.

In individuals with diabetes and low-to-normal BMI, structured nutritional assessment represents an important evaluative domain and may be explored using validated tools such as the Controlling Nutritional Status (CONUT) score, the PG-SGA, or selected biochemical markers (e.g., serum prealbumin), rather than reliance on screening based solely on body size or weight metrics (11, 27, 32, 33).

Assessment of autoimmune markers, including antibodies to glutamic acid decarboxylase 65, insulinoma-associated antigen 2, and zinc transporter 8, may further assist in differentiating T5DM from LADA or late-onset T1DM (2, 3, 30). Key operational discriminators between proposed T5DM and major alternative etiologies of low-BMI or insulin-deficient diabetes presentations are summarized in **Table 2**. Among islet autoantibody-negative individuals, β-cell functional reserve may be assessed using fasting and/or stimulated C-P measurements, providing an indication of endogenous insulin secretory capacity and supporting differentiation from alternative low-BMI diabetes presentations when interpreted alongside clinical features as needed (3, 8, 31, 34).

**Table 2:** High-yield differential diagnosis for lean diabetes phenotypes relevant to the proposed T5DM framework.

Phenotype	Age of onset	Ketosis tendency	Islet autoantibodies	C-P	IR features	Key clues
Proposed T5DM	Adolescence–early adulthood	Rare	Negative	Low–moderate (limited reserve)	Often relatively preserved insulin sensitivity	Nutritional vulnerability; Islet autoantibodies−; C-P low–moderate; exclude alternative etiologies
Lean T2DM	Adulthood	Rare	Negative	Preserved early (normal/high)	IR despite low BMI (NAFLD, acanthosis)	Central adiposity/NAFLD; Islet autoantibodies−; C-P preserved early
T1DM	Childhood–adolescence	Frequent	Positive	Low	Not primary driver	Autoimmune markers; higher DKA risk
LADA	Adulthood	Occasional	Positive	Gradually declining	Variable	Autoimmune markers; delayed insulin requirement
Monogenic (MODY)	Adolescence–early adulthood	Rare	Negative	Preserved	Not primary driver	Young onset; AD family history; Islet autoantibodies−; consider genetics
Pancreatogenic (type 3c/FCPC)	Variable	Variable	Negative	Reduced	Not primary driver	Pancreatitis/FCPC; steatorrhea; low fecal elastase; abnormal imaging

C-P, C-peptide; IR, insulin resistance; NAFLD, non-alcoholic fatty liver disease; AD, autosomal dominant; FCPC, fibrocalculous pancreatic diabetes; LADA, latent autoimmune diabetes in adults; T1DM, type 1 diabetes mellitus; T2DM, type 2 diabetes mellitus. C-P categories reflect relative endogenous insulin secretory capacity (stimulated testing preferred where feasible), and IR features denote clinical surrogates such as central adiposity, acanthosis nigricans, or NAFLD. This table supports exploratory differential evaluation of lean or insulin-deficient diabetes phenotypes; the proposed T5DM row represents a provisional research construct and does not constitute formal diagnostic criteria.

In selected clinical contexts, such as early disease onset, strong familial clustering, or atypical clinical features, targeted genetic testing may provide additional information to support evaluation for MODY (2, 34). Within this research-oriented framework, priority is given to assessment of nutritional status, evaluation of β-cell functional reserve, and exclusion of alternative etiologies; the co-occurrence of nutritional impairment and reduced β-cell reserve in the absence of islet autoimmunity represents a pattern central to the proposed conceptualization of T5DM.

Nutritional impairment in this framework is not defined by a single biomarker. Screening tools (e.g., CONUT, PG-SGA) and selected laboratory indices should be interpreted in clinical context, particularly under conditions of inflammation, infection, hepatic dysfunction, or renal disease. In low-resource settings, pragmatic combinations of clinical history (dietary scarcity, unintentional weight loss), feasible anthropometry/body-composition surrogates, and basic laboratories may be the most implementable approach.

### 3.3 Limitations of low BMI

BMI has historically served as a pragmatic epidemiological surrogate in contexts where overt undernutrition was prevalent; however, its discriminatory value for identifying T5DM is limited in contemporary settings. Low BMI does not reliably distinguish nutrition-conditioned β-cell vulnerability from constitutional leanness and may also reflect genetic background, socioeconomic factors, or secondary catabolic stress during disease progression, thereby reducing its utility as an antecedent or defining marker (3, 4, 35–37).

Beyond its limited phenotypic specificity, BMI provides little insight into β-cell functional reserve, as reflected by C-P secretion, and fails to capture key dimensions of nutritional vulnerability, including skeletal muscle mass and overall nutritional risk profiles (4, 10). In contrast, selected biochemical indices (e.g., serum albumin and lymphocyte count) and measures of body composition and fat distribution have shown associations with nutritional risk and adverse outcomes in diabetes populations, offering complementary prognostic information beyond anthropometric assessment alone (13, 32, 33, 38).

Available evidence supports multidimensional nutritional phenotyping—integrating body composition measures with selected biochemical and inflammatory indices—as a more informative approach for characterizing nutrition-conditioned β-cell vulnerability in T5DM (11, 15).

## 4 Epidemiological characteristics

### 4.1 Global epidemiology

The conceptualization of T5DM has been informed by clinical phenotypes historically described as MRDM and has predominantly been reported among individuals with a history of early-life nutritional deprivation, particularly in resource-limited settings with constrained access to structured endocrine care (3, 5).

The global burden of diabetes continues to rise, affecting an estimated 529 million individuals worldwide in 2021, approximately 96% of whom are classified as having T2DM (39). Despite this expanding burden, international classification and surveillance frameworks, including those of the WHO and the International Classification of Diseases (ICD), do not currently classify T5DM as a distinct diagnostic category. As a result, nutritionally conditioned, insulin-deficient diabetes phenotypes are typically subsumed under existing classifications, which may limit accurate estimation of T5DM prevalence in population-level datasets (3, 5).

In many LMICs, chronic nutritional stress increasingly coexists with a rising burden of metabolic disease, and diabetes has been reported to present among younger individuals with low BMI with histories of dietary scarcity rather than obesity-associated IR (3–5, 36, 37). Such presentations are poorly captured by conventional T2DM frameworks and have prompted growing discussion in relation to the conceptual construct of T5DM (3–5). However, global epidemiological databases remain largely structured around traditional diabetes categories, which may constrain phenotypic resolution and contribute to under-recognition of nutrition-conditioned diabetes phenotypes (1, 3, 5, 39).

Evidence from population-based surveys provides illustrative examples of this potential diagnostic masking. In India, analyses of the National Family Health Survey have shown that a notable proportion of individuals diagnosed with T2DM fall below conventional body size thresholds used to define obesity, particularly among socioeconomically disadvantaged populations (35). Comparable patterns have been reported in East Africa, where studies from Ethiopia describe frequent co-occurrence of malnutrition and endocrine dysfunction among individuals with diabetes, including hypogonadism, findings that may reflect broader nutritional vulnerability rather than obesity-driven metabolic disease (40).

Broader indicators of metabolic vulnerability may be inferred from complication profiles observed across diverse diabetes populations. Diabetes and hypertension remain leading causes of chronic kidney disease globally, with the most rapid increases in diabetes-related kidney disease reported in LMICs, reflecting a high background burden of metabolic stress rather than phenotype-specific risk (41, 42). Additional indirect signals include high rates of anemia among individuals with diabetes in Sri Lanka and severe diabetic retinopathy reported among younger, lean patients in Eastern Europe, patterns that may be associated with reduced metabolic reserve and heightened susceptibility to microvascular complications, although causal relationships remain to be established (1, 5, 43, 44).

Environmental stressors may further influence the broader epidemiological context in which nutritionally vulnerable diabetes phenotypes are observed. Exposure to fine particulate matter (PM_2_._5_) has been associated with increased diabetes incidence at the population level, with potentially greater effects reported in regions characterized by socioeconomic disadvantage and nutritional vulnerability (45). The COVID-19 pandemic similarly underscored the impact of systemic stress on metabolic stability, as infection susceptibility, dysglycemia, and accelerated muscle loss were frequently reported among individuals with diabetes, particularly in nutritionally compromised settings (46).

Epidemiological patterns consistent with the proposed T5DM framework appear to show regional clustering in LMICs, where nutritional vulnerability, environmental stressors, and limited health-care infrastructure intersect. The absence of nutrition-sensitive surveillance frameworks likely obscures accurate estimation of disease burden and constrains development of targeted public health responses, supporting the incorporation of standardized nutritional assessment and body composition measures into population-based surveillance systems (1, 3, 5).

### 4.2 Challenges in low-income countries

Low-income countries (LICs) represent a distinctive public health context in which nutritionally conditioned diabetes phenotypes relevant to T5DM are increasingly discussed, particularly during periods of rapid nutritional and epidemiological transition (3, 5). Historically dominated by protein–energy malnutrition and micronutrient deficiency, many LICs now face a double burden of malnutrition characterized by the coexistence of undernutrition with rising prevalence of overweight, obesity, and T2DM (36, 37, 47).

Within this transitional environment, metabolic vulnerability related to early-life nutritional deprivation may persist despite declining rates of overt undernutrition. Individuals exposed to nutritional stress during critical developmental periods may enter adulthood with constrained β-cell reserve and subsequently develop diabetes predominantly through endocrine insufficiency rather than classical IR when exposed to later metabolic stressors (3, 4). These features are consistent with pathophysiological mechanisms discussed within the conceptual framework of T5DM (3, 5).

In such settings, diabetes phenotypes described in relation to T5DM are often characterized by low-BMI body habitus, impaired β-cell secretory capacity, relatively preserved insulin sensitivity, low propensity for ketoacidosis, and absence of islet autoantibodies—features that distinguish them from autoimmune T1DM and obesity-driven T2DM (3, 4). Evidence from survivors of early-life malnutrition further suggests persistent abnormalities in both exocrine and endocrine pancreatic function, supporting a developmental contribution to long-term pancreatic vulnerability (7). Despite these features, nutritionally conditioned diabetes phenotypes are frequently misclassified as low-BMI T2DM, contributing to underestimation of the epidemiological burden associated with T5DM (5).

Structural constraints further limit accurate identification and management of T5DM in LICs. Access to key diagnostic tools, including glycated hemoglobin (HbA1c), C-P testing, and renal function assessment, is often inconsistent, while diabetes care imposes substantial out-of-pocket costs, particularly for young individuals requiring long-term insulin therapy (48, 49). Limited availability of structured diabetes education, preventive screening, and trained nutrition or metabolic specialists may further contribute to suboptimal glycemic control and the near absence of formal nutritional risk assessment in primary care settings (48–50).

Population-based surveys indicate that many individuals in LMICs meet elevated diabetes risk criteria, yet access to dietary counselling and routine glucose screening remains limited, particularly among socioeconomically disadvantaged groups (51). This gap may be compounded by convergence of infectious and metabolic disease burdens, exemplified by the high prevalence of tuberculosis–diabetes comorbidity, which has been discussed as reflecting underlying immunometabolic vulnerability in contexts characterized by chronic infection and nutritional stress (52).

These factors may create a concealed high-risk environment for T5DM in LICs, in which early-life nutritional deprivation, subsequent metabolic stress, and constrained health-care capacity converge to amplify endocrine vulnerability. Addressing this gap may benefit from nutrition-sensitive surveillance frameworks and integrated care approaches that prioritize assessment of nutritional reserve rather than reliance on weight- or body size–centered metabolic control paradigms alone.

## 5 Nutritional management strategies

### 5.1 Role of nutritional supplementation

Nutritional considerations are increasingly recognized as an important component of diabetes care in individuals with nutritional vulnerability (3, 11). As outlined earlier, T5DM has been discussed in association with chronic nutritional deprivation, constrained β-cell functional reserve, and limited metabolic adaptability, rendering glycemic stability particularly sensitive to nutrient availability and catabolic stress (3, 5).

In this context, nutritional supplementation has been discussed as a supportive consideration rather than a substitute for pharmacological therapy in nutritionally vulnerable diabetes populations (5, 53). Inadequate energy intake, protein deficiency, and micronutrient depletion have been associated with impaired insulin secretion, loss of lean tissue, and broader metabolic vulnerability, highlighting the relevance of nutritional status in shaping glycemic responses alongside glucose-lowering treatment (6, 11).

Across heterogeneous diabetes populations, structured oral nutritional supplements, targeted micronutrient repletion, and nutrition education have been associated with improvements in nutritional markers, lean-mass indices, and selected glycemic endpoints in small trials and real-world cohorts (11, 54–61). In hospitalized and diabetic foot ulcer settings, malnutrition and higher nutritional risk scores predict poorer recovery and adverse outcomes, supporting routine nutritional risk assessment as part of diabetes care (32, 62–64).

These data provide context for considering nutrition-focused support in phenotypes discussed within the T5DM framework, but phenotype-specific interventional evidence remains limited.

### 5.2 Dietary therapy and lifestyle interventions

Lifestyle modification is a core component of diabetes care. When considered in relation to nutritionally vulnerable phenotypes, including those discussed within the conceptual framework of T5DM, emphasis has been placed on approaches that differ from weight-centric strategies typically applied in obesity-driven T2DM management (3). Discussions in this context highlight the importance of preserving skeletal muscle mass, minimizing additional nutritional stress, and supporting metabolic stability (3, 9, 11).

In T2DM, structured lifestyle programs that integrate dietary modification, physical activity, and behavioral support improve glycemic control and cardiometabolic risk markers, with heterogeneity in response across individuals and CGM-derived phenotypes (65–69). For nutritionally vulnerable presentations, discussions emphasize nutrient density, glycemic stability, and preservation of skeletal muscle rather than weight reduction, while noting limited phenotype-specific evidence (3, 9, 11, 70, 71).

Bariatric and metabolic surgical interventions are primarily applied in obesity-driven metabolic disease and are not typically considered in lean or nutritionally vulnerable diabetes phenotypes (72, 73). Accordingly, dietary strategies discussed in relation to phenotypes framed within the T5DM construct have focused on glycemic variability, micronutrient adequacy, and preservation of anabolic substrates rather than weight reduction per se (11).

Overall, lifestyle principles relevant to nutritionally vulnerable diabetes emphasize muscle preservation and avoidance of additional catabolic stress, but require validation within T5DM-oriented cohorts.

### 5.3 Pharmacological response considerations in T5DM

Pharmacological responsiveness in T5DM has been discussed as potentially differing from that observed in classic T2DM, based on underlying pathophysiological distinctions rather than established therapeutic evidence (3). Whereas T2DM is typically characterized by predominant IR with relatively preserved β-cell secretory capacity in early disease stages, conceptual discussions of T5DM emphasize a phenotype marked by constrained endogenous insulin secretory reserve despite relatively preserved peripheral insulin sensitivity (3, 6).

Within this framework, agents that enhance endogenous insulin secretion, including sulfonylureas, have been discussed in relation to potential mechanistic limitations in settings characterized by reduced β-cell reserve (6). In phenotypes where insulin secretory capacity is developmentally constrained, such agents may impose additional demand on residual β-cell mass, raising theoretical considerations regarding durability of glycemic response rather than establishing evidence of differential clinical efficacy.

Incretin-based therapies, including glucagon-like peptide-1 receptor agonists (GLP-1RAs) and dual incretin agonists, lower glycaemia predominantly through glucose-dependent mechanisms and may therefore be mechanistically relevant in phenotypes characterized by limited β-cell reserve (74, 75). However, their net metabolic effects in nutritionally imprinted diabetes phenotypes, including those discussed in relation to T5DM, remain insufficiently defined.

Similarly, the metabolic context of sodium–glucose cotransporter 2 inhibitors (SGLT2is) may plausibly be influenced by underlying nutritional and metabolic factors in nutritionally vulnerable phenotypes, particularly in settings where reduced metabolic reserve or early renal vulnerability coexist (2, 11).

**Figure 4:**
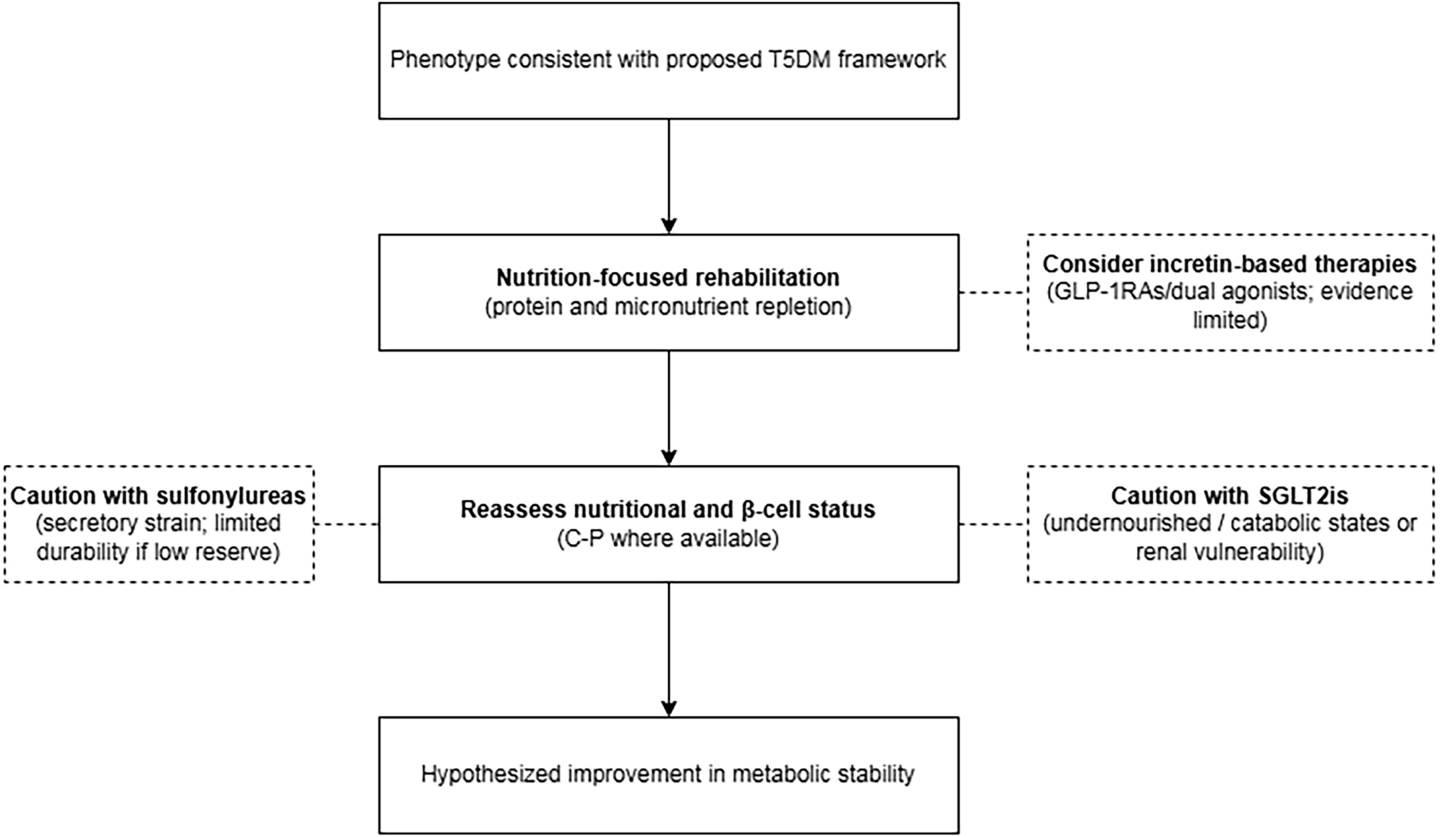
Conceptual, nutrition-informed pharmacometabolic considerations for T5DM. This schematic summarizes a hypothesis-generating, nutrition-integrated framework for pharmacometabolic considerations in phenotypes consistent with the proposed T5DM construct. A central premise is that nutritional reserve and residual β-cell functional capacity may shape pharmacological responsiveness in this setting more than insulin resistance alone. Nutrition-focused rehabilitation (protein and micronutrient repletion) is positioned as a foundational strategy, accompanied by iterative reassessment of nutritional status and β-cell reserve (including C-P where available). Incretin-based therapies (GLP-1 RAs and dual incretin agonists) are shown as mechanistically relevant options, although evidence remains limited in nutritionally imprinted phenotypes. In contrast, secretagogue-based therapies such as sulfonylureas warrant caution where β-cell reserve is low due to potential secretory strain and uncertain durability. SGLT2is are also highlighted as requiring caution in undernourished or catabolic states and in the presence of renal vulnerability. Overall, the framework emphasizes concept-driven, nutrition-informed treatment considerations and indicates the value of dedicated clinical studies to define pharmacological responses across nutritionally conditioned diabetes phenotypes.

Taken together, available conceptual discussions suggest that pharmacological responsiveness within the proposed T5DM framework may be influenced by nutritional reserve and residual β-cell structural capacity than by IR alone (3, 6). Key mechanistic considerations, evidence boundaries, and nutritional risk notes relevant to suspected T5DM are summarized in **Table 3**. Within this hypothesis-generating framework, a nutrition-informed pharmacometabolic perspective may be considered, in which pharmacological responses are considered alongside indices of nutritional status and β-cell reserve rather than weight- or body size–centered treatment heuristics alone. **Figure 4** summarizes this conceptual framework, illustrating how nutritional reserve and β-cell functional capacity may interact to influence pharmacological considerations.

**Table 3:** Therapeutic considerations in suspected T5DM: evidence grading and nutritional boundaries.

Drug class	Mechanistic rationale in T5DM	Evidence level	Nutritional risk	Clinical caution
Metformin	Hepatic glucose suppression with minimal β-cell demand	Moderate	Weight loss; vitamin B12 reduction	Interpret within a nutrition-informed framework; avoid unintended weight loss
SGLT2is	Insulin-independent glucose lowering	Moderate	Volume depletion; euglycemic DKA risk	Consider nutritional/metabolic context and renal vulnerability; monitor volume status
Incretin-based therapies	Glucose-dependent enhancement of residual insulin secretion	Weak	Appetite suppression; lean mass loss	Net effects in nutritionally conditioned phenotypes remain undefined; monitor nutritional status
Sulfonylureas	Direct stimulation of limited β-cell reserve	Weak	Secretory strain if reserve is low	Potential limitations in low-reserve states; durability considerations
Basal insulin	Exogenous insulin replacement in insulin-deficient states	Strong	Hypoglycemia risk if intake is low	Use with explicit nutritional assessment and β-cell evaluation; titrate to intake

SGLT2is, sodium–glucose cotransporter 2 inhibitors; DKA, diabetic ketoacidosis. Evidence grading categories are defined as follows: None, no relevant clinical evidence; Weak, limited or indirect clinical evidence; Moderate, consistent evidence from controlled trials in related diabetes populations; Strong, established efficacy in insulin-deficient diabetes populations, not specific to T5DM. Evidence levels refer to data derived from related diabetes phenotypes rather than from trials conducted in confirmed T5DM cohorts. Drug classes are ordered according to increasing dependence on endogenous or exogenous insulin signaling. This table summarizes mechanistic considerations and safety boundaries in suspected T5DM and does not constitute therapeutic recommendations. No randomized controlled trials have prospectively stratified pharmacotherapy by nutritional reserve in this phenotype.

## 6 Future research directions

### 6.1 Research methods and data-driven classification

Recent advances in diabetes research increasingly employ data-driven approaches to refine disease classification beyond traditional clinical phenotypes (31, 76). Large-scale cohort studies and machine learning–based clustering analyses have revealed substantial heterogeneity within conventionally defined T1DM and T2DM, motivating multidimensional precision subtyping models that integrate clinical features, metabolic traits, genetic information, and longitudinal outcomes (31, 34, 74, 76, 77). These developments have indirect relevance to metabolically atypical or underrepresented phenotypes, including those discussed within the exploratory framework of T5DM.

To achieve more granular subclassification, recent studies have incorporated electronic health record (EHR) data, genotype-derived polygenic risk scores (PRSs), metabolomic profiles, and longitudinal glucose trajectories into unsupervised pipelines, enabling identification of diabetes subgroups with distinct metabolic trajectories (31, 34, 76, 77). Population-scale EHR–survey–linked algorithms, including those developed within the All of Us Research Program, have improved case ascertainment and alignment with genetic risk profiles compared with EHR-only approaches (78), while PRS-based frameworks have enhanced subtype discrimination in multi-ancestry cohorts, particularly for autoimmune diabetes (79). However, these models remain predominantly weighted toward immune-mediated and adiposity-related dimensions and do not systematically incorporate developmental nutritional exposure, body composition, or constrained β-cell functional reserve.

Continuous glucose monitoring–based clustering further extends data-driven phenotyping by leveraging high-resolution glycemic dynamics. Multilevel analyses have identified CGM-derived subgroups differing in glycemic variability alongside paired indices of β-cell function and insulin sensitivity (80). While informative, CGM-centered frameworks primarily capture glucose–insulin dynamics and, by design, do not incorporate indicators of nutritional reserve, catabolic stress, or early-life nutritional deprivation. Similar limitations apply to data-driven subphenotyping across the prediabetes continuum and autoimmune diabetes, where improved prediction of progression, complications, and mortality has been reported, yet frameworks remain anchored in metabolic excess or immune activity rather than nutritional depletion (81, 82).

These advances highlight a methodological gap: current precision-classification frameworks underrepresent nutritional reserve depletion and nutrition-conditioned β-cell vulnerability, features proposed as central within the conceptual framework of T5DM. Addressing this gap will require nutrition-aware phenotyping strategies and explicit integration of nutritional and developmental determinants into future classification models.

### 6.2 Further clinical trials

Although the importance of nutrition in diabetes management is increasingly acknowledged, substantial gaps remain in interventional evidence addressing nutrition-related endocrine dysfunction, particularly for phenotypes discussed within the conceptual framework of T5DM. Most data linking malnutrition to adverse diabetes outcomes derive from observational studies in heterogeneous populations (e.g., hospitalized individuals and patients with chronic kidney or cardiovascular disease), in which impaired nutritional status is consistently associated with increased mortality, prolonged hospitalization, suboptimal glycemic control, and higher complication burden (11, 13, 83, 84). However, these studies were not designed to distinguish nutrition-conditioned phenotypes from obesity-associated T2DM, limiting causal inference and mechanistic specificity. In addition, longitudinal outcome data specific to historical MRDM cohorts and populations phenotypically consistent with the proposed T5DM framework remain scarce, highlighting the need for prospective studies incorporating predefined clinical endpoints.

To date, no randomized controlled trials have been explicitly designed to evaluate nutrition-stratified or nutrition-tailored pharmacotherapy within the proposed T5DM research framework. As a result, optimal therapeutic sequencing and safety in nutritionally conditioned, insulin-deficient phenotypes remain empirically guided rather than evidence-defined (3).

Existing trials in predominantly overweight or nutritionally heterogeneous T2DM cohorts (e.g., meal-replacement or intermittent-fasting strategies, diabetes-specific oral nutritional supplements, and structured self-management programs) show improvements in HbA1c and related outcomes, but rarely incorporate standardized malnutrition phenotyping or long-term endpoints, limiting translation to T5DM-oriented questions (56, 85–87). Across studies, nutritional risk has been assessed using non-uniform tools (e.g., CONUT, PNI, SGA), which limits cross-study comparability and trial harmonization (27, 33, 87). Emerging data also suggest interactions between malnutrition and systemic inflammation, supporting integrated outcome frameworks beyond glycaemia alone (15).

### 6.3 Conceptual considerations for nutrition-stratified pharmacological trial design within the proposed T5DM framework

Future randomized controlled trials investigating phenotypes discussed within the conceptual framework of T5DM could incorporate nutritional reserve as an explicit stratification dimension, aligning trial architecture with hypothesized mechanisms in which chronic nutritional deprivation, impaired anabolic capacity, and constrained β-cell functional reserve play central roles (3, 11). Participants could be prospectively stratified using feasible tools such as the CONUT score and the PG-SGA, complemented by selected biochemical markers (e.g., serum albumin or prealbumin) and measures of muscle mass and function, with recognition of context dependence in inflammatory or catabolic states (9, 11, 27, 33, 88).

Within each nutritional stratum, pharmacological strategies could be evaluated as monotherapy or combined with structured nutritional support. Candidate interventions may include GLP-1RAs, dual incretin receptor agonists (e.g., tirzepatide), SGLT2is, or basal insulin–based strategies, selected according to trial objectives and safety considerations (74, 75, 89). Such designs would enable exploratory testing of whether pharmacological responsiveness varies as a function of nutritional reserve and β-cell functional capacity.

Outcome frameworks relevant to phenotypes discussed within the T5DM framework may require composite measures extending beyond HbA1c alone, incorporating β-cell reserve (e.g., fasting and stimulated C-P AUC), glycemic variability, preservation of lean muscle mass, biomarkers of inflammatory and catabolic stress, and longitudinal trajectories of nutritional recovery (8, 9, 11, 13, 15, 80). Secondary analyses could examine whether improvements in nutritional reserve modify pharmacodynamic responses, supporting exploratory construction of a nutrition–pharmacology response map to inform sequencing hypotheses without prescriptive claims.

If supported by empirical evidence, such a framework may strengthen biological characterization of phenotypes conceptualized within the T5DM construct and enable more precise therapeutic investigation.

## 7 Conclusion

T5DM, as discussed in recent international consensus statements (3, 5, 6), should not be interpreted as the introduction of a novel nosological category, but rather as a harmonized research construct aimed at integrating historical descriptions of MRDM with contemporary discussions of undernutrition-associated, insulin-deficient diabetes. While ICD-11 provides administrative coding relevant to malnutrition-associated diabetes (21), biological boundaries, reproducible operational criteria, and validated diagnostic thresholds remain insufficiently defined. Accordingly, T5DM should presently be regarded as a provisional, hypothesis-generating phenotype within precision diabetes research.

A central limitation is persistent diagnostic ambiguity. No standardized framework yet defines the degree of nutritional depletion or β-cell functional limitation required for classification. Low BMI alone does not reliably distinguish nutrition-conditioned β-cell insufficiency from low-BMI T2DM, latent autoimmune diabetes in adults, pancreatogenic diabetes, or monogenic forms. Without structured evaluation of nutritional reserve, endogenous insulin secretion, and autoimmune status, misclassification remains likely.

The implications of such misclassification extend beyond semantics. Insulin-deficient, nutritionally vulnerable individuals may be subsumed under obesity-centric T2DM paradigms, potentially obscuring underlying pathophysiology and limiting the interpretability of epidemiological and interventional research within the proposed T5DM framework. Incorporation of standardized nutritional assessment and β-cell reserve evaluation may therefore enhance cohort characterization without prematurely redefining global diagnostic taxonomies.

Therapeutically, evidence remains insufficient to support phenotype-specific treatment algorithms for T5DM. No randomized trials have prospectively stratified pharmacological interventions according to nutritional reserve in populations phenotypically consistent with the proposed T5DM framework. Future investigations should integrate predefined nutritional stratification, multidimensional metabolic endpoints, and longitudinal outcome assessment to determine whether nutrition-conditioned β-cell vulnerability confers distinct therapeutic trajectories.

Until such data are available, T5DM should remain positioned as a structured research hypothesis within evolving precision-classification systems, rather than as a formally established clinical entity.
